# Raman-based PAT for multi-attribute monitoring during VLP recovery by dual-stage CFF: attribute-specific spectral preprocessing for model transfer

**DOI:** 10.3389/fbioe.2025.1631807

**Published:** 2025-08-21

**Authors:** Annabelle Dietrich, Luca Heim, Jürgen Hubbuch

**Affiliations:** Institute of Process Engineering in Life Sciences, Section IV: Biomolecular Separation Engineering, Karlsruhe Institute of Technology (KIT), Karlsruhe, Germany

**Keywords:** Raman spectroscopy, virus-like particles, cross-flow filtration, process analytical technology, partial least squares regression, spectral preprocessing, process monitoring, detector oversaturation

## Abstract

Spectroscopic soft sensors are developed by combining spectral data with chemometric modeling, and offer as Process Analytical Technology (PAT) tools powerful insights into biopharmaceutical processing. In this study, soft sensors based on Raman spectroscopy and linear or partial least squares (PLS) regression were developed and successfully transferred to a filtration-based recovery step of precipitated virus-like particles (VLPs). For near real-time monitoring of product accumulation and precipitant depletion, the dual-stage cross-flow filtration (CFF) set-up was equipped with an on-line loop in the second membrane stage. With this set-up, spectral data from three CFF runs were collected, differing in initial product concentration and process parameters. Under the scope of multi-attribute monitoring, a comprehensive investigation of the sensor sensitivity towards protein and precipitant and their Raman spectral features was carried out. This study reveals much higher sensitivity towards the precipitant ammonium sulfate (AMS) than the VLPs and the need for attribute-specific spectral preprocessing. To enhance the detector’s sensitivity towards proteins, a higher exposure time was applied during CFF processing than during model building from pure-component stock solutions. As a result of this increased exposure time, the predominant sulfate band exhibited oversaturation effects, which otherwise could have been used for AMS quantification via linear regression. Nevertheless, AMS prediction using purpose-driven preprocessing operations and PLS models was achieved with normalization and a data-driven variable selection technique, next to baseline correction and signal smoothing. For VLP monitoring, a novel pre-cropping approach improved spectral appearance after further preprocessing in protein-associated wavenumber regions. However, fluctuations in prediction were much higher for VLPs than for AMS, and prediction accuracy was especially limited in low protein concentration ranges. These results highlight the potential of Raman-based PAT sensors for real-time monitoring of biopharmaceutical processes, while underscoring the general importance of attribute-specific selections of sensors, preprocessing operations, and models for PAT tool development.

## 1 Introduction

Virus-like particles (VLPs) have emerged as a promising alternative to viral vectors, with applications ranging from vaccines to drug and gene delivery systems (Qian et al., 2020). Structurally mimicking native viruses but lacking viral genetic material, VLPs offer a unique combination of safety and efficacy (Chackerian, 2007; Zeltins, 2013; Nooraei et al., 2021). In vaccine application, their higher immunogenicity compared to subunit vaccines (Tariq et al., 2022) can be even further directed or enhanced by surface modifications using genetic or chemical approaches (Chung et al., 2020). Since Hepatitis B core Antigen (HBcAg) VLPs were expressed (Burrell et al., 1979) and visualized (Stahl et al., 1982) as one of the first VLPs, they continue to be the subject of ongoing research and recent advancements have been achieved in surface displays (Moradi Vahdat et al., 2021; Hassebroek et al., 2023) or payload packaging (Cooper and Shaul, 2005; Porterfiled et al., 2010; Petrovskis et al., 2021).

Due to the diverse structural complexity of different VLP types, purification strategies are usually developed individually, which may lead to costly manufacturing processes involving numerous unit operations (Moleirinho et al., 2020). The need for broadly applicable, scalable, and cost-effective manufacturing processes drives the development of novel purification strategies (Effio and Hubbuch, 2015). Due to the relatively large size of the VLPs, processes based on size-selective separation techniques such as precipitation or filtration exhibit standardized platform characteristics and provide an alternative to chromatographic methods (Hillebrandt and Hubbuch, 2023). Using cross-flow filtration (CFF), buffer exchange by constant-volume diafiltration (DF) enables dynamic processes previously achieved only through dialysis or dilution, while also allowing product concentration by ultrafiltration (UF) (van Reis and Zydney, 2007). Recent developments have demonstrated the applicability of CFF throughout downstream processing of HBcAg VLPs, from the initial capture step for VLP re-dissolution from VLP precipitates (Hillebrandt et al., 2020; Dietrich et al., 2025), to the final polishing steps including their disassembly into subunits (Hillebrandt et al., 2021) and subsequent reassembly into capsids (Rüdt et al., 2019). These developments position filtration-based purification technologies at the forefront of standardized platform technologies for protein nanoparticle purification.

Filtration set-ups typically include in-line flow and pressure sensors to monitor and control standard process parameters such as transmembrane pressure and permeate flux (van Reis and Zydney, 2007). However, gaining further insights into such dynamic processes typically relies on manual sampling and off-line analytics, limiting the scope of process understanding and resulting in product loss, especially in small-scale unit operations. In 2004, the FDA formally established the framework for Process Analytical Technology (PAT) to support enhanced process understanding, monitoring, and control by measuring process parameters and product quality attributes (FDA, 2004). Through sensor integration and evaluation of the collected data, process data can be continuously gathered in (near) real-time (Rathore et al., 2010; Glassey et al., 2011). In filtration set-ups, sensors are implemented directly in-line or within an on-line measurement loop. For the monitoring of quality attributes in biopharmaceutical filtration processes, several soft sensors have been recently developed by coupling spectroscopic sensors and chemometrics, including ultraviolet-visible (UV/Vis) (Rüdt et al., 2019; Rolinger et al., 2020a; Hillebrandt et al., 2022), mid-infrared (MIR) (Wasalathanthri et al., 2020), near-infrared (NIR) (Thakur et al., 2020; 2021; Vaskó et al., 2024), and Raman spectroscopy (Rolinger et al., 2023; Vaskó et al., 2024). These spectroscopic sensors differ in their underlying measurement principles and inherent sensitivity to specific substances. While UV/Vis spectroscopy is highly accurate for protein concentrations and has already been used to monitor product variants (Brestich et al., 2018) and quaternary structure (Rüdt et al., 2019; Hillebrandt et al., 2022), the simultaneous monitoring of protein and excipient concentrations can be realized by MIR (Wasalathanthri et al., 2020), NIR (Thakur et al., 2021), or Raman spectroscopy (Weber and Hubbuch, 2021; Rolinger et al., 2023). For Raman spectroscopy, recent advancements have been made towards monitoring of particulates in phase-behavior dependent processes, such as crystallized enzymes (Wegner et al., 2024) or precipitated VLPs (Dietrich et al., 2024), as well as monitoring of multiple quality attributes during fermentation (Santos et al., 2018), chromatography (Wang et al., 2023), and formulation (Wei et al., 2022) of monoclonal antibodies.

Given the high sensitivity of Raman spectroscopy, raw Raman spectral data exhibit undesired variability, requiring considerable effort in data preparation before being used for modeling. Such pre-processing operations comprise signal correction techniques to correct baseline, background, or scattering effects, filter techniques to reduce uncorrelated noise or extract spectral features by derivative-filtering, and cropping techniques to reduce dimensions or focus on relevant spectral regions (Rinnan et al., 2009; Bocklitz et al., 2011). Beyond manual selection of cropping intervals, ranging from solely discarding the edge regions to selecting spectral regions of interest, variable selection techniques offer data-driven selection, aiming to minimize the loss of important spectral data while improving model robustness (Andersen and Bro, 2010). Variable importance in projection (VIP) represents such a data-driven strategy, quantifying the contribution of each wavenumber to partial least squares (PLS) models (Mehmood et al., 2012).

In many studies, however, a sequence of preprocessing operations with their parameters is given for a model presented, with limited in-depth analysis of Raman spectral features beforehand or explanations for choosing those operations. An approach addressing systematic soft sensor development was reported by Dietrich et al. (2024), who first studied the effects of selected preprocessing operations on Raman spectral data before screening multiple combinations of preprocessing operations, so-called preprocessing pipelines, to assess the impact of individual operations on model performance. Although they demonstrated the quantification of selectively precipitated VLPs in crude, clarified lysate through incorporation of specific preprocessing operations to account for turbidity and eliminate interferences caused by contaminating species, they reported limited transferability of the VLP models from off-line screening to on-line fed-batch data. Model transfer may be more successful in process stages with increased product purity, such as in the recovery step of these precipitated VLPs after reducing the impurity load.

Seamless VLP recovery is enabled by integrated dual-stage CFF, isolating the re-dissolved VLPs through precipitant depletion in the second membrane stage (Dietrich et al., 2025). Here, Raman spectroscopy has already been used for off-line quantification of the precipitant, ammonium sulfate (AMS), but so far, no attempt has been made to develop multi-attribute monitoring to provide simultaneous insights into VLP enrichment and AMS depletion.

In this study, we present a systematic, purpose-driven approach for PAT tool development for multi-attribute monitoring by Raman spectroscopy. The development aims for simultaneous insights into product accumulation and precipitant depletion during a filtration-based recovery step of precipitated VLPs using the integrated, dual-stage CFF set-up proposed by Dietrich et al. (2025). First, we investigate the contributions of product and precipitant to the spectral data using stock solutions of the pure components. Based on these insights and aiming to ultimately transfer the developed models to process data containing contributions of both species simultaneously, the effects of individual preprocessing operations on the Raman spectral data are thoroughly assessed. We develop regression models of varying complexity using either product- or precipitant-containing stock solutions and attribute-specific spectral preprocessing operations, thereby addressing challenges such as differences in detector sensitivity and detector saturation effects. By implementing Raman spectroscopy in an on-line loop in the second membrane stage of the dual-stage CFF setup, we collect process data in near real-time from three CFF experiments with variations in initial product concentration and process parameters. Eventually, we transfer the developed models to on-line process data to visualize the process dynamics of VLP recovery and precipitant depletion and demonstrate the importance of individual preprocessing operations for model transfer.

## 2 Materials and methods

### 2.1 Virus-like particles

The VLP of interest assembles of C-terminally truncated wild-type HBcAg proteins (Cp149), for which the plasmids were initially provided by Prof. Adam Zlotnick from Indiana University (Zlotnick et al., 1996). The procedure of their intracellular expression in *Escherichia coli* (*E.coli*), cell harvest, cell lysis, and lysate clarification was performed as described in Hillebrandt et al. (2020). All clarified lysate material was pooled to create a single batch for all experiments. Clarified lysate was stored in aliquots at −20 °C and thawed on the day of the experiments, followed by sterile filtration and conditioning for immediate use. Conditioning involved diluting the clarified lysate with pH 8.0 lysis buffer (50 mM Tris, 100 mM NaCl, 1 mM EDTA) to achieve a specific ultraviolet (UV) absorbance (EXP1–2) or spiking with VLP-enriched material (EXP3), and adjusting to 0.25% (v/v) polysorbate 20 for all experiments (EXP1–3). Note that the spiking (EXP3) was meant to match the level of host-cell impurities in the EXP1–2 material, so the spiking material replaced the amount of dilution material initially needed. The conditioning of clarified lysate is summarized for all experiments in Table 1. The VLP-enriched material was derived from the final product of EXP2, which was further dialyzed into the lysis buffer overnight using a 10 kDa MWCO Slide-A-Lyzer G2 cassette (Thermo Fisher Scientific Inc., Waltham, US).

**TABLE 1:** Experimental conditions of the three CFF experiments (EXP1–3).

Experiment	Clarified lysate	Process	Process monitoring		
	Condition	DV	On-line loop flow rate	Acquisition mode	Raman exposure time
	-	-	mL min^−1^	-	ms
EXP1	dilution	6	0.6	semi-continuous	175/1,250
EXP2	dilution	7	1.2	continuous	1,250
EXP3	spike	7	1.2	continuous	1,250

### 2.2 Capture process and process monitoring

Fully integrated processing was enabled using the dual-stage CFF setup presented by Dietrich et al. (2025) with minor modifications. With this dual-stage CFF set-up, the VLP capture process involves selective VLP precipitation, followed by two consecutive, constant-volume DF steps for washing the VLP precipitates (DFI) and final recovery of the re-dissolved VLPs (DFII/UF). Precipitation and washing were similarly performed for all experiments according to Dietrich et al. (2025), while several settings during VLP recovery (DFII/UF) differ between the experiments EXP1 and EXP2–3, as summarized in Table 1.

**FIGURE 1:**
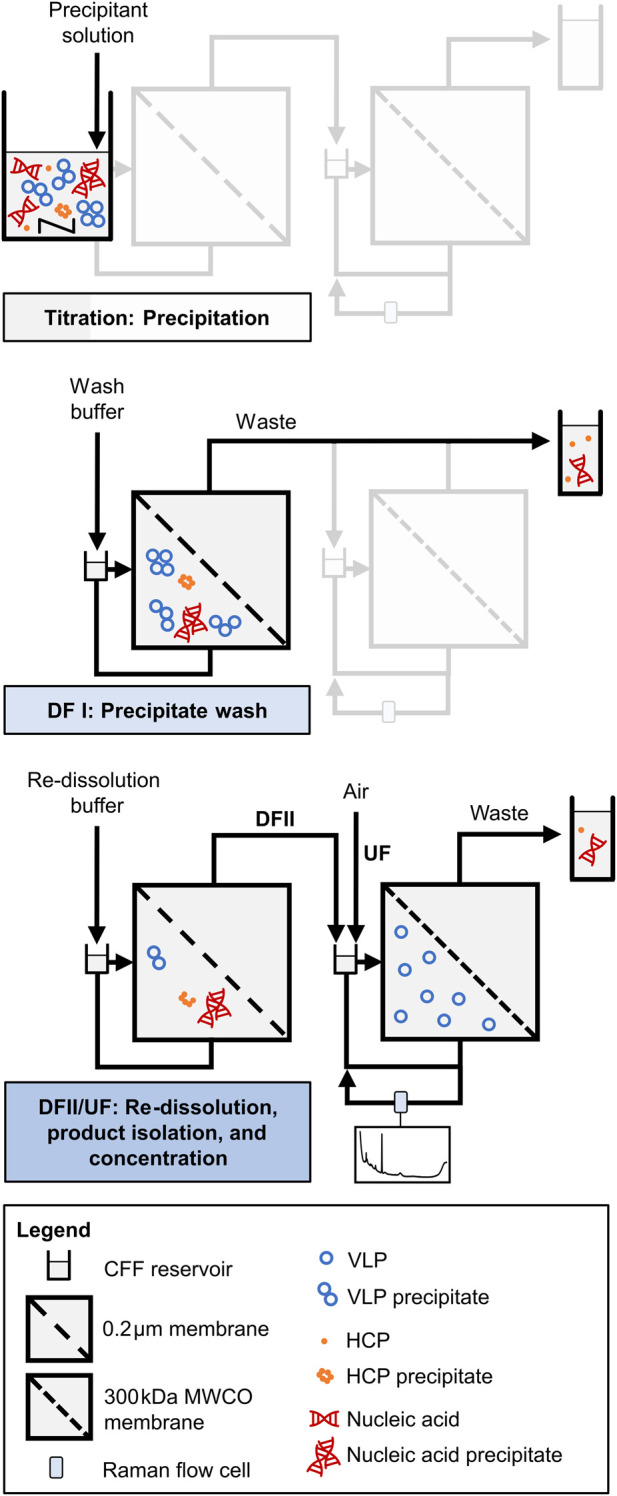
Schematic illustration of VLP processing by integrated dual-stage CFF. A dual-stage CFF set-up with a 0.2 µm/300 kDa MWCO membrane configuration is used for the process steps precipitation, precipitate wash (DFI), and VLP recovery (DFII/UF). VLP recovery involves DF-induced VLP re-dissolution, VLP isolation in the second membrane stage, and VLP concentration by subsequent UF. An on-line loop equipped with a Raman flow cell in the second membrane stage allows for near real-time monitoring by Raman spectroscopy. Adapted from Dietrich et al. (2025).

All consecutive process steps are illustrated schematically in Figure 1 and a piping and instrumentation diagram is additionally provided in Supplementary Figure S1. Two serially connected KrosFlo Research KRIIi CFF units (Spectrum Labs, Rancho-Dominguez, US) were equipped with 0.2 µm and 300 kDa MWCO Hydrosart membranes (200 cm^2^; Sartorius Stedim Biotech GmbH, Göttingen, DE), respectively. The permeate flow rates were controlled at 2 mL min^−1^ by an in-house developed, MATLAB-based backpressure valve controller, involving automatic backpressure valves (Spectrum Labs) in the retentate streams and SLS-1500 flow sensors (Sensirion AG, Stäfa, CH) in the permeate streams. An ÄKTA Start (Cytiva, Uppsala, SE) connected in series enabled permeate stream monitoring by in-line UV and conductivity sensors and collecting permeate stream fractions by the fraction collector. Valves were included in the setup to bypass the second CFF unit in the wash step (DFI). An on-line loop was further installed in the second CFF unit, including a Minipuls 3 peristaltic pump (Gilson, Villiers le Bel, FR), and a flow cell for Raman measurements. The on-line loop flow rate was set to 0.6 mL min^−1^ (EXP1) or 1.2 mL min^−1^ (EXP2–3).

Selective VLP precipitation was performed in the reservoir of the first CFF unit, which was induced by gradually adding the precipitant stock solution (4 M AMS) to the conditioned, clarified lysate until reaching the target precipitant concentration of 1.1 M AMS. Following a 30-min incubation under stirring conditions, the wash step (DFI) was carried out, and the permeate bypassed the second CFF unit to monitor and collect the permeate stream directly. The VLP precipitates were washed with wash buffer (lysis buffer containing 1.1 M AMS) for 6 to 6.5 diafiltration volume (DV), until the UV absorbance of the permeate stream dropped below 60 mAU to ensure that the majority of still soluble impurities passed the 0.2 µm membrane. It has to be noted that the conductivity data have been qualitatively used as an indicator for the presence of AMS during the wash step (data not shown).

The VLPs were recovered in the second DF step (DFII) with pH 7.2 re-dissolution buffer (50 mM Tris, 150 mM NaCl) for six (EXP1) or seven DVs (EXP2–3) using the dual-stage CFF setup. DF induced VLP re-dissolution, the re-dissolved VLPs passed the 0.2 µm membrane and accumulated in the second CFF retentate, as they are not able to pass the 300 kDa MWCO membrane of the second CFF unit. By decoupling the first CFF unit, the accumulated VLPs were further concentrated from 25 mL (DV) to a final volume of 10 mL by integrated UF. During this VLP recovery (DFII/UF), process monitoring was performed by semi-continuous (EXP1, alternating exposure times: 175 and 1,250 ms) or continuous (EXP2–3, 1,250 ms exposure time) Raman measurements in the implemented on-line loop to obtain on-line spectral data. Further, process samples for off-line analysis were taken at 0.5 DV, at each DV, and the final UF step. Off-line Raman measurements at 175 ms and 1,250 ms were performed on each process sample to obtain off-line spectral data, alongside off-line UV spectroscopy to quantify the VLP content.

### 2.3 Stock solutions for model building

AMS-containing stock solutions were prepared by proportionally mixing wash buffer and re-dissolution buffer to mimic the DF dynamic in the VLP recovery step (DFII) and hence fully cover the buffer composition and AMS content (0–1.1 M AMS). Raman spectra were recorded off-line at 110 and 175 nm exposure times and used for model building.

The VLP stock solution was derived from the final product of the dual-stage CFF process presented in the study by Dietrich et al. (2025), which was further concentrated by UF using Vivaspin 20 centrifugal filters (Sartorius Stedim Biotech GmbH). A dilution series of the VLP stock solution using the re-dissolution buffer was prepared and off-line measured by Raman spectroscopy at an exposure time of 1,250 ms. The spectral data were used for model building.

### 2.4 Analytics

#### 2.4.1 Raman spectroscopy

The Raman spectrometer HyperFlux™ PRO Plus 785 (Tornado Spectral Systems, Toronto, CA) was equipped with a BioReactor BallProbe within a flow cell (both MarqMetrix, Seattle, US) and controlled by SpectralSoft 3.2.6 (Tornado Spectral Systems). The spectra were recorded in the spectral range from 200 to 3300 cm^−1^ with 1 cm^−1^ resolution, a laser power of 495 mW, and exposure times of 175 or 1,250 ms. For off-line Raman measurements, the flow cell was equipped with inlet and outlet capillaries and manually filled with the sample using a syringe.

#### 2.4.2 UV spectroscopy

The UV spectrometer consisted of an RS diode array detector integrated into a high performance liquid chromatography (HPLC) system, all controlled by Chromeleon 6.8 (Dionex Ultimate 3000 RS, Sunnyvale, US). Size-exclusion chromatography (SEC) using a BioSEC-5 column (4.6 × 300 mm, 5 μm, 1,000 A; Agilent, Santa Clara, US) was used to separate differently sized species with method settings similar to Hillebrandt et al. (2020): 20 µL injection volume, 0.4 mL min^−1^ flow rate, and 14 min isocratic elution. The UV spectra were recorded in the spectral range from 220 to 400 nm. With peak areas at 280 nm, a universal purity measure regarding host-cell proteins (HCPs) and nucleic acids derived by dividing A280_VLP_ by A280_total_ and is described as SEC-purity. A280_VLP_-derived VLP concentrations were calculated using Beer’s law and a theoretical Cp149 extinction coefficient of 1.764 g L^−1^ (ProtParam tool; Gasteiger et al. (2005)).

### 2.5 Data analysis and computation

Data analysis and computation were performed in Python 3.8. Different strategies were used for spectral data preprocessing and regression modeling for AMS and VLP quantification. Model building was exclusively performed with off-line spectral data derived from stock solutions. The evaluated error metrics included the root mean squared error (RMSE) and the coefficient of determination $\left(R^{2}\right)$ to assess model accuracy.

#### 2.5.1 Spectral data processing and model building—AMS

Spectral data preprocessing covered averaging, normalization, baseline correction, smoothing, and cropping. Averaged spectra from 50 recordings were normalized using the OH Raman band at 3299 cm^−1^ to account for turbidity effects and variations in applied exposure times. A Whittaker filter employing the adaptive smoothness penalized least squares (asPLS) (Zhang et al., 2020) was applied for baseline correction ( λ value of $6\times 10^{7}$ , second-order difference matrix, tolerance of $1\times 10^{-3}$ ), followed by a Savitzky-Golay filter (SGF) for spectral smoothing (second-degree polynomial, window size of 11). Three cropping strategies were applied to account for selected features attributed to AMS. The wavenumber 980 cm^−1^ reflecting the highest intensity was used for a linear regression model (LR_AMS_). Unaffected by edge effects from prior baseline correction, the spectral interval 340–2650 cm^−1^ was selected for a PLS model (PLS_AMS_). To qualitatively assess the importance of specific wavenumbers and identify AMS-associated regions, VIP scores were applied according to Mehmood et al. (2012). The resulting spectral intervals, 427–471, 600–634, 960–999, and 1103–1115 cm^−1^, were scaled to unit variance and subsequently used for regression modeling for refined PLS models (PLS-VIP4_AMS_, PLS-VIP2_AMS_).

Spectra recorded at 175 ms exposure time were used for model building. For both PLS models, hyperparameter optimization with the number of latent variables in the range of 2–10 was performed by cross-validation using a random split of 80% training data and 20% validation data. The NIPALS algorithm was applied according to Wold et al. (2001). For all regression models, spectra recorded at 110 ms exposure time were used as test data.

#### 2.5.2 Spectral data processing and model building—VLP

Spectral data preprocessing included averaging, pre-cropping, baseline correction, smoothing, and cropping. Two pre-cropping (P1/P2) and cropping (C1/C2) intervals were combined, resulting in four differently preprocessed spectra for model building (PLS-PX-CY_VLP_). Averaged spectra from 50 recordings were first pre-cropped by excluding the wavenumber ranges between 920 and 1030 cm^−1^ (P1) or between 920 and 1200 cm^−1^ (P2), which includes the region with the highest AMS-associated intensity. Baseline correction was performed by employing the Whittaker filter ( λ value of $1\times 10^{9}$ , third-order difference matrix, tolerance of $1\times 10^{-4}$ ), followed by SGF-based spectral smoothing. Further, the spectra were cropped to the interval 1203–1349 cm^−1^ (C1) or 1331–1349 cm^−1^ (C2). Hyperparameter optimization and model building were performed, as described in Section 2.5.1 but spectra recorded at 1,250 ms exposure time were used as test data.

## 3 Results

### 3.1 AMS: Raman spectroscopy and linear regression for precipitant quantification

Raman spectra of AMS-containing stock solutions were recorded over the precipitant concentration range of 0–1.1 M AMS covering the range for VLP recovery by CFF. Spectral data recorded at 175 ms exposure time were used for preprocessing pipeline development and model building. Table 2 summarizes the parameter settings for spectral preprocessing operations and model building.

**TABLE 2:** Spectral preprocessing and model building.

Model	Normalization	Pre-cropping	Baseline		Smoothing		Cropping	Hyperparameter
	Wavenumber	Wavenumber	Lambda	Derivative	Window	Polynom	Wavenumber	Number of components
	cm^−1^	cm^−1^					cm^−1^	-
LR_AMS_	3299	-	6e-7	2	11	2	980	-
PLS_AMS_	3299	-	6e-7	2	11	2	[340, 2650]	2
PLS-VIP4_AMS_	3299	-	6e-7	2	11	2	[427,471],[600,634],[960,999],[1103,1115]	2
PLS-VIP2_AMS_	3299	-	6e-7	2	11	2	[427,471],[1103,1115]	2
PLS-P1-C1_VLP_	-	[920, 1030]	6e-9	2	11	2	[1203, 1349]	2
PLS-P1-C2_VLP_	-	[920, 1030]	6e-9	2	11	2	[1331, 1349]	2
PLS-P2-C1_VLP_	-	[920, 1200]	6e-9	3	11	2	[1203, 1349]	2
PLS-P2-C2_VLP_	-	[920, 1200]	6e-9	3	11	2	[1331, 1349]	2

**FIGURE 2:**
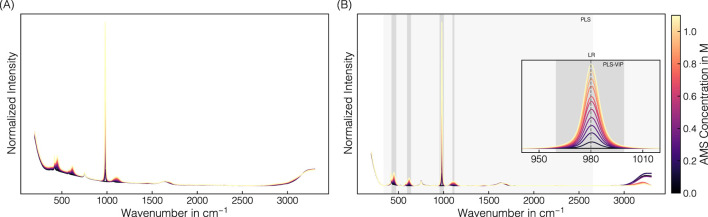
Raman spectral data: AMS. Derived from a set of stock solutions with varying AMS concentrations, averaged raw spectral data **(A)** were preprocessed by normalization, baseline correction, and smoothing **(B)**. The predominant Raman band near 980 cm^-1^ corresponding to the sulfate ion is used for linear regression. The PLS model includes the wavenumber interval 340–2650 cm^-1^ highlighted in light-gray, while the VIP-based intervals selected for the PLS-VIP models are shaded gray. The spectra are colored with brighter colors representing higher AMS concentrations.

The spectral preprocessing pipeline involved normalization, baseline correction, and signal smoothing derived from the pipeline development of Dietrich et al. (2025) to remove baseline drifts and enhance spectral differences. Figure 2 illustrates raw and preprocessed spectra of the AMS-containing stock solution set over the entire spectral range. In the raw spectra, precipitant-dependent baseline drifts are visible with baseline increases with higher AMS concentrations (cf. Figure 2A). Baseline correction using the asPLS Whittaker filter, combined with smoothing using the SGF filter, consistently removed these baseline drifts across the entire recorded wavenumber range (cf. Figure 2B). The distinct resolution of the predominant Raman band near 980 cm^−1^ is attributable to gradually increasing sulfate ions of AMS (Spinner, 2003; Fontana et al., 2013). In general, several components of precipitant and buffer contribute to the spectral appearance, which is described in Supplementary Section 2 and presented in a higher resolution in Supplementary Figure S2.

The uniformly preprocessed spectra have been cropped to a distinct wavenumber or wavenumber interval prior to regression modeling. Besides linear regression using the predominant sulfate-associated band maximum at 980 cm^−1^, several PLS models were evaluated, differing in the cropped wavenumber intervals used for model building (cf. Table 2; Figure 2B). Simple linear regression aligned well for the test set with a $R^{2}$ of 0.999 and a RMSE of 0.013 M AMS over the concentration range from 0 to 1.1 M AMS. It has to be noted that cross-validated PLS models using the entire spectral range or selected wavenumber intervals identified through VIP scores showed comparable error metrics, with similar $R^{2}$ values and RMSE ranging between 0.010 and 0.013 M AMS. Interestingly, the VIP scores applied to qualitatively assess the importance of specific wavenumbers identified higher contributions of sulfate-associated than ammonium-associated regions. Further, scaling to unit variance improved error metrics for PLS-VIP_AMS_ models, but resulted in higher errors for a PLS model build with scaled spectral intensities ( $R^{2}$ : 0.990, RMSE: 0.035 M) than the presented PLS_AMS_ model without scaling ( $R^{2}$ : 0.999, RMSE: 0.012 M). Due to distinct sulfate Raman bands, noise-dominated regions may be mistakenly weighted as important in the scaled PLS model with noise-induced variations in areas lacking true signal.

In summary, simple spectral preprocessing followed by linear regression using the intensity at 980 cm^−1^ enables Raman spectroscopy for AMS content quantification. Spectral comparison suggests model transferability to CFF-based processes without buffer or protein species interference.

### 3.2 AMS: on-line precipitant quantification by PLS-VIP model transfer despite different exposure times and detector saturation effects

**FIGURE 3:**
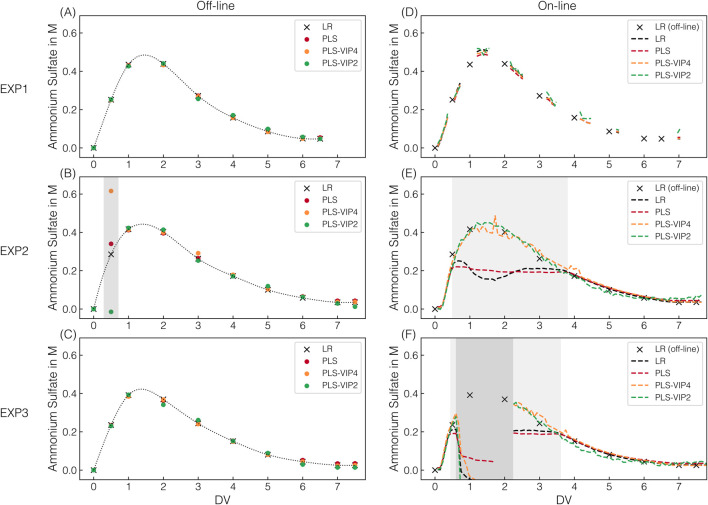
AMS model predictions. For all CFF runs, the predicted AMS concentrations are shown, which derived from individual off-line measurements at 175 ms exposure time **(A**–**C)** and on-line measurements **(D**–**F)** in semi-continuous (175 ms, EXP1) or continuous (1,250 ms, EXP2–3) acquisition mode; along with their corresponding color assignments for the models used. Dotted lines (off-line) using a quadratic fit serve solely as visual guides to facilitate interpretation. The dashed lines (on-line) represent continuous prediction. Predictions by defective spectra are highlighted with dark-gray shaded areas. Predictions by spectra with oversaturation of the 980 cm^-1^ band at 1,250 ms exposure time are shaded light-gray.

All the AMS models built on stock solutions were transferred to process-derived spectra to determine the AMS depletion throughout the CFF-based recovery step of the re-dissolved VLPs (DFII/UF). Figure 3 presents the predicted AMS concentrations for the applied AMS models on the off-line and on-line spectral data for the three CFF experiments (EXP1–3) performed.

Across all CFF experiments, the observed progression of the AMS concentration follows a distinct pattern, with first increasing and then, from the second DV onward, decreasing AMS concentrations. This progression in the second membrane stage is attributable to the process step (DFII). DF with re-dissolution buffer in the dual-stage CFF set-up leads to an overall AMS depletion present in the first membrane stage, resulting in an overlap of AMS accumulation and simultaneous AMS depletion in the second membrane stage. Comparable AMS progressions observed within the first six DVs of the DFII process indicate consistent and reproducible processing by dual-stage CFF. Extending the DFII process from six (EXP1) to seven DVs (EXP2–3) further reduced the AMS content in the final retentate before the subsequent UF, representing an improvement in the overall VLP recovery process.

All AMS models applied on off-line spectral data recorded at 175 ms Raman exposure time show comparable AMS content predictions at the sampling points (cf. Figures 3A–C). With only one exception, the predictions of the PLS models fluctuate only marginally and without a distinct pattern around the prediction obtained using linear regression. However, all PLS-based AMS content predictions for the 0.5 DV sample in EXP2 deviate significantly from those of the linear regression (cf. Figure 3B). Those observed deviations in prediction can be attributed to spectral appearance as PLS models incorporate additional spectral intervals beyond the 980 cm^−1^ band maximum used for linear regression. Since both under- and overestimations are observed, a generally defective spectrum has been suspected and identified (cf. Supplementary Figure S3A). Overall, simple linear regression relying on the 980 cm^−1^ band intensity of preprocessed Raman spectra was successfully transferred to process-derived spectra for off-line AMS quantification.

For on-line AMS quantification, the on-line spectra derived from either semi-continuous (175 ms, EXP1) or continuous (1,250 ms, EXP2–3) spectral acquisition were assessed regarding AMS predictions (cf. Figures 3D–F). In the semi-continuous spectral acquisition mode during EXP1, spectra were continuously recorded in time frames around the sampling points using the same Raman exposure time of 175 ms as for off-line AMS quantification.

The PLS-VIP2_AMS_ model predictions exhibit marginal fluctuations within those time frames compared to the more consistent predictions of all other models (cf. Figure 3D). However, those consistent and to off-line quantification comparably precise predictions provide a solid basis for continuous process monitoring in near real-time.

**FIGURE 4:**
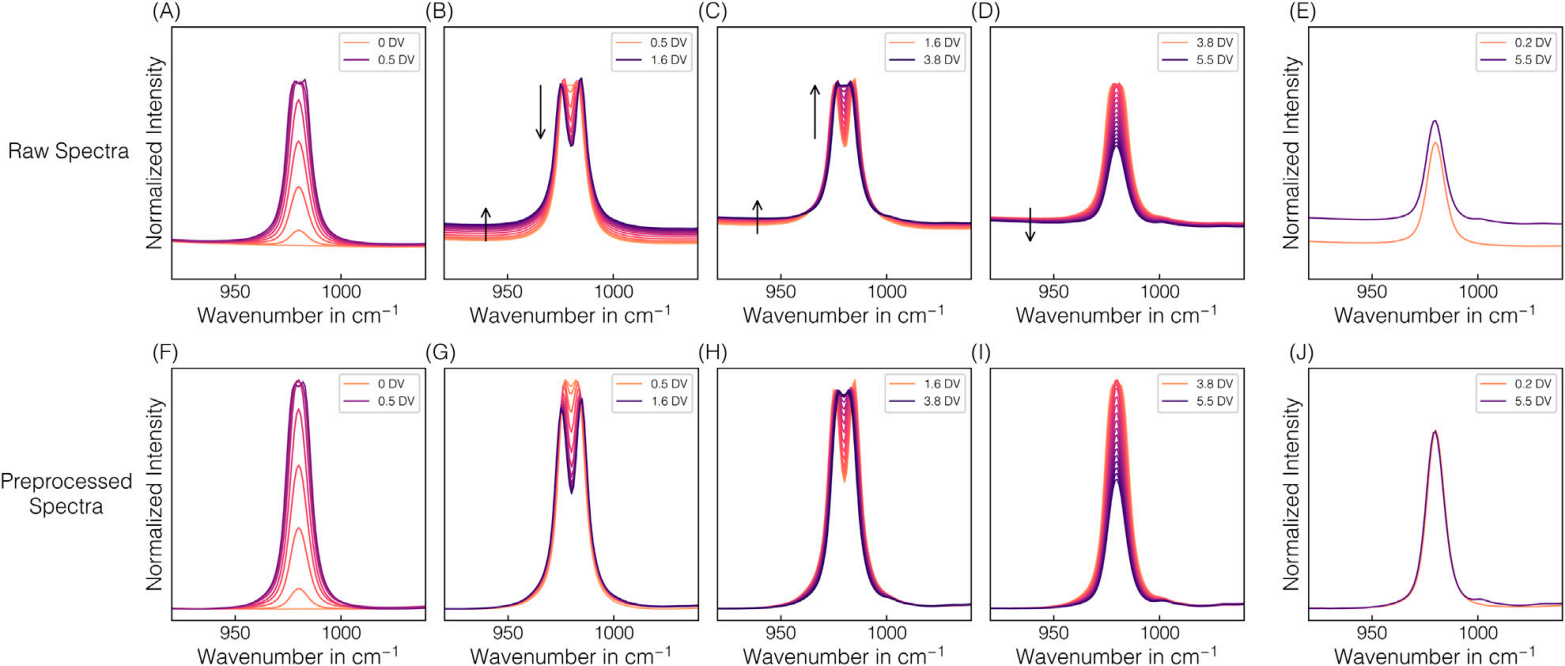
Spectral oversaturation effects. The changes in spectral appearance of the predominant 980 cm^-1^ sulfate band in the raw **(A**–**E)** and preprocessed **(F**–**J)** Raman spectra from EXP2 on-line Raman measurements are depicted and resolved by DV in panels to visualize the spectral effects of oversaturation: reaching saturation after 0.5 DV **(A**,**F)**, remaining in a oversaturated state due to the still increasing AMS concentration until 1.6 DV **(B,G)** and a further decreasing AMS concentration **(C,H)** until reaching the AMS concentration at 3.8 DV after which the system falls below saturation again **(D,I)**. Arrows serve as visual guides to highlight the formation or decay of the split peak depending on the AMS concentration in the saturated state **(B,C)** and baseline shifts **(B**–**D)**. Additionally, two spectra obtained at 0.2 DV and 5.5 DV at identical AMS concentrations are shown **(E**,**J)**.

Given that a higher exposure time of 1,250 ms is required for the later model transfer for simultaneous VLP prediction, continuous spectral acquisition at 1,250 ms was performed during EXP2–3. The higher applied exposure time led to an oversaturation of the predominant 980 cm^−1^ sulfate band, resulting in a distinctive appearance of the corresponding band region. Exemplarily illustrated for EXP2, Figure 4 shows raw and preprocessed spectral data of the 980 cm^−1^ sulfate band region, resolved by DV in panels to visualize the spectral effects of oversaturation. The greater the oversaturation with higher AMS concentrations until 1.6 DV, the more pronounced the resulting split peak appears, and the more distinct the baseline shift towards higher intensities is observed (cf. Figure 4B). With afterwards decreasing AMS concentrations and the corresponding reformation of the split peak, the baseline shifts slightly further towards higher intensities, contrary to the expectation (cf. Figure 4C). Only later in the process is a slight baseline shift towards lower intensities observed (cf. Figure 4D), but the baseline no longer reaches its initial level. The difference in the baseline level is exemplified by two spectra with identical AMS concentration but recorded at different DVs (cf. Figure 4E). As expected, this difference in the baseline level is no longer apparent after preprocessing (cf. Figure 4J), as is the case for all previously described baseline shifts (cf. Figures 4G–I). The unexpected behavior of the baseline shift suggests the influence of a secondary factor unrelated to AMS concentration.

The split peak appearance is reflected in the incorrect predictions between 0.5 and 3.6–3.8 DVs when applying the linear regression and the PLS_AMS_ model (cf. Figures 3E,F). These two models can only reliably predict the AMS concentration as long as saturation does not occur, which corresponds to approximately 0.2 M AMS as critical AMS concentration at the exposure time of 1,250 ms. As expected, the progression of the predicted AMS concentration during oversaturation using linear regression directly reflects the split peak behavior at 980 cm^−1^. In contrast, the two other presented PLS-VIP models are indeed capable of predicting AMS concentrations higher than 0.2 M AMS despite the observed spectral appearance (cf. Figures 3E,F). While the PLS-VIP4_AMS_ model predictions exhibit minor fluctuations in the time frame of band oversaturation, the predictions below 0.2 M AMS are fairly consistent and comparable to those of the PLS_AMS_ or linear regression model. It has to be noted that besides the PLS-VIP4_AMS_ model using all VIP-selected wavenumber intervals attributed to sulfate contributions, model building with a combination of either three or two intervals have shown comparable error metrics during model building using 175 ms exposure time where no band oversaturation was present. However, except for the wavenumber combination of the PLS-VIP2_AMS_ model, all failed in prediction accuracy when applied to EXP2–3 data derived from on-line Raman spectral data at 1,250 ms exposure time (data not shown), even though the saturated band interval was excluded. The PLS-VIP2_AMS_ model demonstrates more stable predictions in the time frame of band oversaturation. However, compared to all other models, it exhibits slightly shifted predictions below 0.2 M AMS towards lower or higher AMS concentrations within the range of 0.2 to 0.08 M AMS or at the final stages of the process, respectively.

Notably, defective spectra were recorded from 0.7 to 1.8 DV in EXP3, exhibiting immense baseline shifts (cf. Supplementary Figure S3B), ultimately leading to false predictions (cf. Figure 3F). Only manually decoupling the on-line loop, flushing it with re-dissolution buffer, and reconnecting it provided expected spectral appearances from 2.2 DV onward.

In summary, the process-derived spectra define the required models for AMS quantification. Although differences in spectral appearance existed as the exposure times varied between model building and process-derived, continuously recorded Raman spectral data, the progression of AMS depletion throughout the CFF-based recovery step (DFII/UF) could be continuously monitored through precise adjustment and refinement of the models using VIP scores.

### 3.3 VLP: spectral pre-cropping improves further spectral preprocessing and PLS model building

Raman spectra of VLP-containing stock solutions were recorded over a VLP concentration range of 0–2.2  gL^−1^. Spectral data recorded at 1,250 ms exposure time were used for preprocessing pipeline development and model building. Table 2 summarizes the parameter settings for spectral preprocessing operations and model building.

**FIGURE 5:**
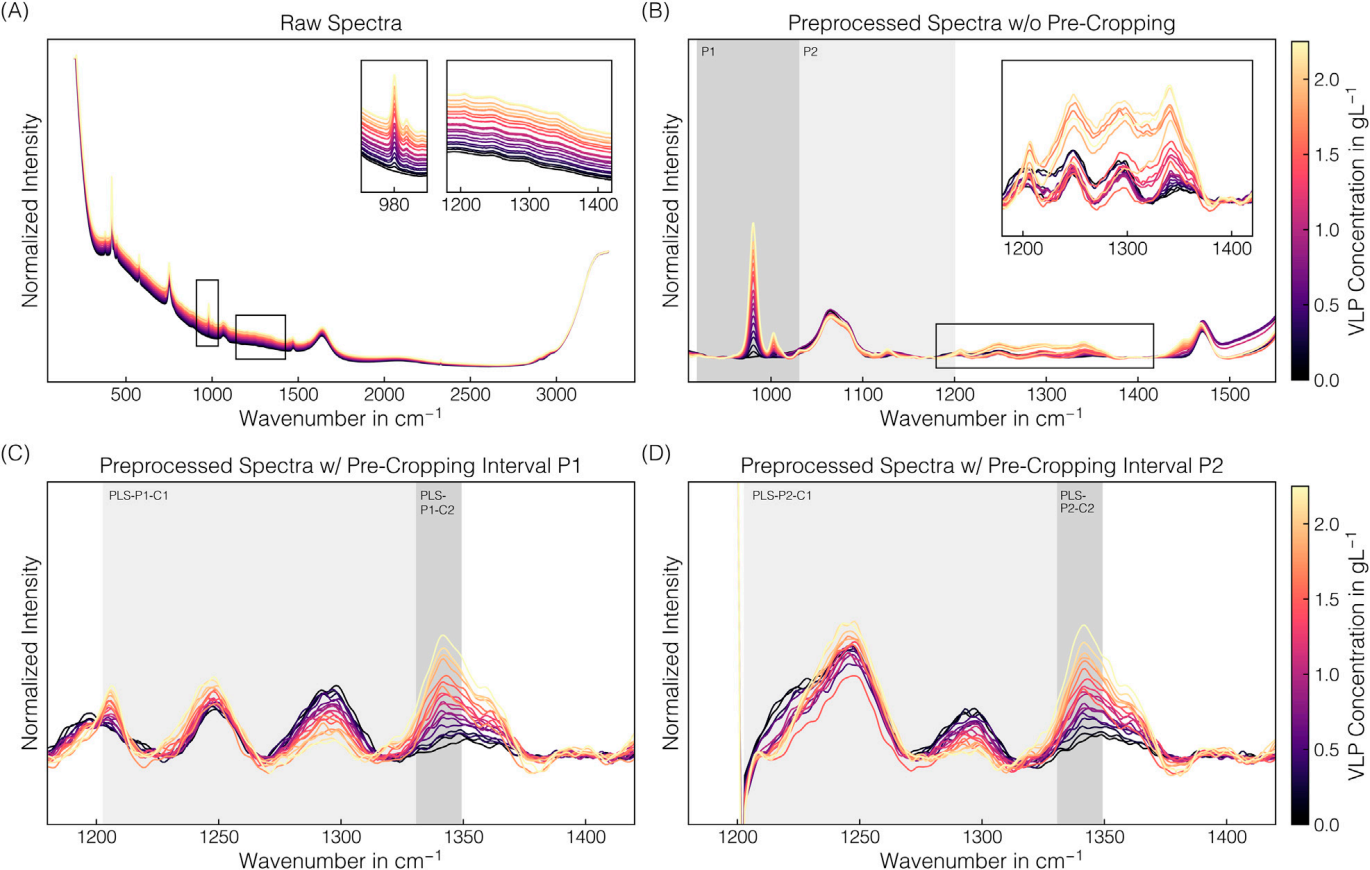
Raman spectral data: VLP. Derived from a set of stock solutions with varying VLP concentrations, averaged raw spectral data **(A)** were differently preprocessed **(B**–**D)**. For raw spectra **(A)** and baseline-corrected, signal-smoothed spectra **(B)**, the wavenumber region around the predominant Raman band near 980 cm^−1^ and the wavenumber region associated with proteins are additionally presented on an enlarged scale. The wavenumber intervals 920–1030 cm^−1^ (P1) or 920–1200 cm^−1^ (P2) removed by pre-cropping prior to preprocessing are highlighted in gray **(B)**. Including pre-cropping changed the spectral appearance in the protein-associated region, as presented in **(C)** and **(D)**, respectively. Pre-cropped, baseline-corrected, and signal-smoothed spectra were further cropped to the wavenumber intervals 1203–1349 cm^−1^ (C1) or 1331–1349 cm^−1^ (C2) for PLS modeling, as highlighted in gray **(C**,**D)**. The spectra are colored with brighter colors representing higher VLP concentrations.

The spectral preprocessing pipeline involved pre-cropping, baseline correction using the asPLS Whittaker filter, and signal smoothing using the SGF filter to remove baseline drifts and enhance spectral differences. Figure 5 illustrates raw and preprocessed spectral data of the VLP-containing stock solution set over the entire spectral range or selected wavenumber intervals. In the raw spectra, baseline drifts are visible with baseline increases with higher VLP concentrations (cf. Figure 5A). By simple baseline correction and signal smoothing without the pre-cropping step beforehand, these baseline drifts could not be consistently removed in the wavenumber region 1200–1400 ^−1^ (cf. Figure 5B). Additionally, the spectra show the 980 cm^−1^ band attributable to sulfate ions (Spinner, 2003; Fontana et al., 2013), indicating residual AMS in the VLP stock solutions deriving from its preparation. As the pronounced phenylalanine band at 1004 cm^−1^ and other protein-associated wavenumber regions 600–880 cm^−1^ and 1200–1800 cm^−1^ (Maiti et al., 2004; Rygula et al., 2013) will be partly obscured when a considerable amount of the precipitant AMS is present, 1200–1400 cm^−1^ region, being not affected by AMS obscuration, was chosen for further preprocessing and model development.

A pre-cropping strategy was introduced, removing selected wavenumber intervals of the spectrum to account for the baseline shifts in this protein-associated region. The wavenumber interval 920–1030 cm^−1^ (P1) was used to eliminate the contributions of the predominant 980 cm^−1^ sulfate band, resulting in a more consistent spectral appearance of preprocessed spectra, allowing trends in the 1200–1400 cm^−1^ region to be observed (cf. Figure 5C). The Raman band at 1206 cm^−1^ is attributed to tyrosine, the band at 1249 cm^−1^ originates from the polypeptide backbone, and the band at 1341 cm^−1^ is a composite of overlapping signals from both the polypeptide backbone and tryptophan (Maiti et al., 2004; Rygula et al., 2013). The band at 1249 cm^−1^ originates from the buffer component Tris (Socrates, 2004). A larger pre-cropping interval of 920–1200 cm^−1^ (P2) to further account for the broad sulfate band around 1106 cm^−1^ resulted in a similar spectral appearance in the 1270–1400 cm^−1^ region but essentially obscured the 1206 cm^−1^ tyrosine band (cf. Figure 5D). Both preprocessed spectra differing in the pre-cropping interval (P1/P2) have been further cropped to a distinct wavenumber interval prior to regression modeling (cf. Table 2; Figures 5C,D). The error metrics $R^{2}$ and RMSE of the PLS-P2-C2_VLP_ model are with 0.999 higher and 0.02  gL^−1^ lower than PLS-P2-C1_VLP_ with 0.994 and 0.05  gL^−1^, respectively. In contrast, both models with the smaller pre-cropping interval (P1) achieved $R^{2}$ values of 0.984 and RMSE values of 0.08  gL^−1^.

In summary, spectral preprocessing was developed through spectral comparison considering (i) the spectral appearance in the protein region and (ii) potential interferences from the precipitant to make the model suitable for data from CFF-based processes. The combination of pre-cropping to remove certain wavenumber intervals, baseline correction, signal smoothing, and further cropping to select intervals in the protein-associated region allows for uniform spectral preprocessing and model building for VLP quantification.

### 3.4 VLP: on-line Raman spectral data reveal VLP accumulation and sensor fouling

**FIGURE 6:**
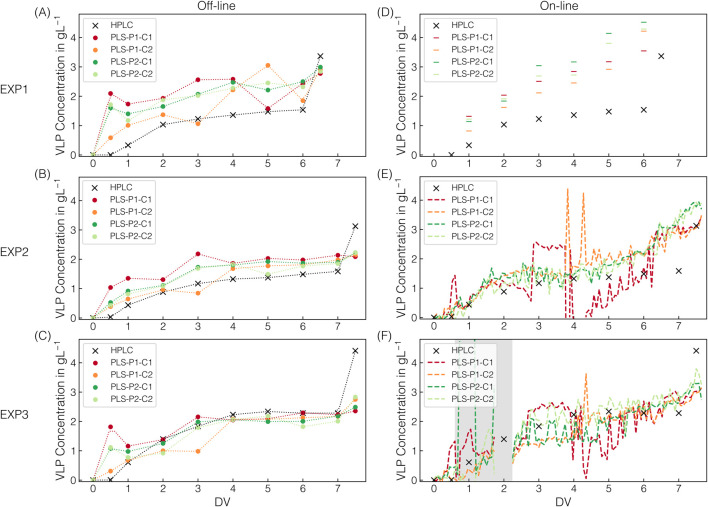
VLP model predictions. Next to HPLC-derived VLP concentrations, the predicted VLP concentrations are shown for all CFF runs. The predictions derived from individual Raman measurements at 1,250 ms exposure time. Predictions from off-line and on-line measurements in semi-continuous (EXP1) or continuous (EXP2–3) acquisition mode are shown in **(A**–**C)** and **(D**–**F)**, respectively, along with their corresponding color assignments for the models used. The models differ in pre-cropping and cropping operations, with their respective intervals P1/P2 and C1/C2. Dotted lines (off-line) serve solely as visual guides to facilitate interpretation. The dashed lines (on-line) represent continuous prediction. Predictions by defective spectra are highlighted with dark-gray shaded areas.

All the PLS_VLP_ models built on stock solutions were transferred to process-derived spectra to determine the accumulation of re-dissolved VLPs in the second membrane stage throughout the CFF-based recovery step (DFII/UF). The PLS_VLP_ models differ in the pre-cropping (P1/P2) and cropping (C1/C2) intervals used in the respective preprocessing operations. Figure 6 presents the predicted VLP concentrations for the applied VLP models on the off-line and on-line spectral data for the three CFF experiments (EXP1–3) performed.

Across all CFF experiments, the observed progression of the HPLC-derived VLP concentration follows a distinct pattern, attributable to the CFF-based VLP recovery step (DFII/UF). DF with re-dissolution buffer in the dual-stage CFF set-up leads to VLP re-dissolution in the first membrane stage, their passage through the microfiltration membrane, and their accumulation in the second membrane stage. DF is followed by UF, resulting in an approximately twofold concentration of the VLPs in the second membrane stage. The higher VLP concentrations observed in EXP3 compared to EXP1–2 are attributable to the VLP-enriched lysate used as starting material for EXP3, representing a diversification of the process data. Final SEC-purity values of the concentrated VLPs ranged between 94% and 96%, consistent with the purity values reported by Dietrich et al. (2025), demonstrating reproducible processing by dual-stage CFF.

Applying the PLS_VLP_ models to off-line spectral data, the trend observed in the HPLC-derived data is reflected in all of the model predictions. However, the prediction accuracy varies between and within the experiments EXP1–3 (cf. Figures 6A–C). In general, the predictions for EXP3 are slightly scattered around the observed VLP concentrations. On the contrary, the ones for EXP2 lie slightly above, seeming to be systematic, and the predictions for EXP1 are significantly higher and exhibit a broader spread. Such a process-dependent occurrence of these deviations can be linked to underlying process-specific factors, resulting in deviating and inconsistent spectral features. When comparing the model predictions for EXP2–3, the predicted VLP concentrations of the PLS-P2 models are almost identical, while those of the PLS-P1 models show higher or lower predicted VLP concentrations at specific DVs (cf. Figures 6B,C). This observation suggests that using the pre-cropping interval P2 results in more consistent spectral features after further spectral preprocessing and less dependence on the cropping interval (C1/C2). Notably, the most noticeable deviations between the HPLC-derived and the predicted VLP concentrations are observed in the range of relatively low and high VLP concentrations at the beginning and the end of the CFF processes, respectively. Overall, the PLS-P2 models show consistent predictions in the range of moderate protein concentrations but lack accuracy at low and high protein concentrations, especially during the concentration step with protein concentrations up to twice as high.

A similar pattern in prediction accuracy emerges when the models are applied to on-line spectral data (cf. Figures 6D–F). For EXP1, fouling on the Raman probe was observed after on-line spectral data acquisition, which, however, did not impact the prediction of AMS (cf. Section 3.2). The deviations in the predictions of the VLP concentration suggest that there was a gradual accumulation of protein on the probe throughout the process, leading to the increasing overestimation of the VLP concentrations (cf. Figure 6D). Concerning fouling, the flow rate of the on-line loop was doubled from EXP1 to EXP2–3, which, along with the switch to continuous spectral data acquisition, represents a process adjustment. For EXP2–3, all predictions seem to scatter around the observed VLP concentrations, with even more pronounced scatter spikes for the PLS-P1 than the PLS-P2 models (cf. Figures 6E,F). Further and consistent with the off-line data predictions, the models also fail to predict the concentration step based on the on-line spectral data. Especially for EXP2, the observed gradual increase in VLP concentration from the fifth DV onward is not reflected by the off-line data (cf. Figure 6E), which may also be indicative of fouling.

No difference in accuracy was observed for predictions from both off-line and on-line spectral data, regardless of whether the sulfate peak at 980 cm^−1^ in the raw spectra was saturated, which the pre-cropping operation was intended to address.

In summary, PLS_VLP_ models were transferred to continuously monitor the accumulation of re-dissolved VLPs in the second membrane stage throughout the CFF-based VLP recovery step (DFII/UF). The PLS-P2 models show the most consistent predictions in the range of moderate protein concentrations, both for off-line and on-line spectral data, across all processes where fouling behavior was neither observed nor suspected.

## 4 Discussion

### 4.1 Sensor selection and implications for multi-attribute monitoring

Raman spectroscopy was selected for multi-attribute monitoring during recovery of precipitated VLPs by dual-stage CFF—a dynamic DF process isolating the re-dissolved VLPs through precipitant depletion in the second membrane stage (Dietrich et al., 2025). Sensitivity and selectivity significantly differed when comparing precipitant or protein monitoring using Raman spectroscopy and chemometrics.

Precipitant quantification is not routinely performed during small-scale screenings with predefined precipitant conditions where the results can be directly correlated. However, its quantification becomes essential in dynamic processes due to varying precipitant concentrations throughout these processes. While Barros Groß and Kind (2018) calculated the theoretical precipitant content based on the volume reduction through evaporative crystallization, Dietrich et al. (2024) further combined the theoretical content with Raman spectral data derived from fed-batch precipitation and chemometrics to predict AMS contents in unseen fed-batch precipitation processes. Accordingly, Dietrich et al. (2024) had already demonstrated the use of Raman spectroscopy for near real-time AMS monitoring through PLS modeling.

With stock solutions covering the AMS concentration range, simple spectral processing, and linear regression using the predominant sulfate band at 980 cm^−1^ (Spinner, 2003; Fontana et al., 2013), Raman spectroscopy is highly selective for AMS quantification. Linear regression has already been applied for off-line AMS quantification to reveal integrated AMS depletion, representing one advantage for VLP recovery by the dual-stage CFF compared to the single-stage CFF set-up (Dietrich et al., 2025). Our study demonstrates the successful transfer of a linear regression model for AMS quantification to process-derived, on-line spectral data, under the condition that no band oversaturation is present, thereby extending its use beyond prior off-line applications. Further, model development for model transfer to accommodate spectral data exhibiting oversaturation effects is successfully demonstrated.

Although conductivity and density measurements, which are both influenced by salts like AMS, also enable real-time monitoring (Rolinger et al., 2021; Hillebrandt et al., 2022), these techniques may lack selectivity, which can compromise accuracy in processes with varying environmental concentrations or compositions. Particularly throughout this dual-stage CFF process for VLP recovery, changes occur not only in precipitant content but also in buffer composition, protein composition, and total protein concentration. Moreover, relying solely on univariate signals from conductivity or density measurements is insufficient for simultaneously predicting both precipitant and product concentrations. Other techniques for AMS quantification pose similar challenges in selectivity and are further limited to off-line measurements as multiple steps are involved. Among others, ammonium is traditionally quantified spectrophotometrically through complex formation (Krug et al., 1979; Patton and Crouch, 1977), while sulfate can be determined fluorescence-based (Saini and Kumar, 2013).

Considering polyethylene glycol (PEG), the other widely used precipitant, using Raman spectroscopy for quantification may pose challenges due to its suspected contributions overlapping with protein-associated wavenumber regions (Kuzmin et al., 2020), which is why enhanced spectral processing and models of higher complexity, such as PLS or non-linear models, may be required. It has to be noted that using PEG in such filtration-based processes in general may have disadvantages, particularly concerning its influence on viscosity and, consequently, filtration behavior (Plisko et al., 2016; Li and Zydney, 2017; Burgstaller et al., 2019) as well as its larger molecular size compared to salts, which may hinder its depletion in the here presented dual-stage CFF process.

Among protein quality attributes, protein concentration is one of the most monitored during the downstream processing of biopharmaceuticals. Several spectroscopic methods and their applicability to protein monitoring are outlined in detail by Rolinger et al. (2020b), among which Raman and UV spectroscopy are sensitive for aromatic amino acids, peptide bonds, and disulfide bonds. Although water interference in Raman spectroscopy is relatively low (Rolinger et al., 2020b), a limited sensor applicability was found at low protein concentrations, attributable to the increasing dominance of the water band. Moreover, the protein predictions exhibit much higher fluctuations than the precipitant predictions, which might result from the substantially lower intensity of protein contributions than sulfate contributions. Raman spectroscopy has recently been compared with UV spectroscopy for predicting monoclonal antibody concentrations in Protein A chromatography, highlighting the significantly superior prediction accuracy of UV spectroscopy (Rolinger et al., 2021). The authors suggest that UV spectroscopy would likely have been more accurate for protein concentration monitoring, which would have resulted in a multimodal spectroscopy setup in this study. Raman spectroscopy has already been implemented together with UV for monitoring enzyme crystallization in complex lysate (Wegner et al., 2024) and as a basis for data fusion to improve prediction accuracy (Rolinger et al., 2021). In such multi-sensor setups, however, the different spectroscopic data require distinct data preprocessing, and, if combined, additional preprocessing may be needed due to signal dispersion between detectors (Rolinger et al., 2020b).

Sensor fouling is a known but rarely reported challenge in spectroscopic process monitoring, describing unintended material accumulation or burning by the laser light. After the first CFF run, spectral inconsistencies were observed in the protein-associated wavenumber region during off-line analysis of process samples, suggesting sensor fouling on the sensor surface or within the flow cell. Although sapphire surfaces and convex geometries tend to be less favorable for material deposition (Prasad et al., 2023), the observed fouling may indicate the influence of residence time within the flow cell. Doubling the flow rate, thereby reducing the residence time by half, prevented the occurrence of spectral inconsistencies in the data of the following CFF runs. In filtration processes, spectroscopic sensors are frequently implemented on-line (Rüdt et al., 2019; Rolinger et al., 2020a; Hillebrandt et al., 2022) or in-line (Wasalathanthri et al., 2020; Thakur et al., 2020; Rolinger et al., 2023; Vaskó et al., 2024) using a flow cell, providing precise control over measurement conditions. Installing sensors directly *in situ* by immersing them into the well-stirred process solution within the system’s reservoir (Wasalathanthri et al., 2020; Thakur et al., 2021; Wegner et al., 2024) may offer a practical alternative to mitigate fouling. Further, the authors suggest that *in situ* monitoring may be less prone to spectra diverging from the others—termed ‘defective spectra’ in this study.

In summary, spectroscopic sensors should be selected based on their sensitivity and selectivity towards the target quality attributes to be monitored. Moreover, sensor implementation should be carefully considered to ensure reliable spectroscopic measurements.

### 4.2 Effects of detector saturation on raw Raman spectral data

The contributions of precipitant and protein to the spectral data were investigated using stock solutions of pure components. A substantially higher sensor sensitivity towards precipitant than protein was observed concerning the spectral features observed in the raw spectral data. The initial objective involved increasing the exposure time to enhance the sensor’s sensitivity to proteins, which, however, led to oversaturation of the predominant sulfate band at 980 cm^−1^ (Spinner, 2003; Fontana et al., 2013) directly related to the precipitant. To the best of the author’s knowledge, the analysis and use of spectral data exhibiting saturation effects at specific wavenumber regions has not been reported in the literature yet.

The raw on-line spectral data collected during processing show a characteristic split peak formation, which stands in contrast to reported oversaturation characteristics observed in the low wavenumber regions, where entire bands disappear due to the baseline being elevated to the saturation level (Tornado, 2021). The manufacturer recommends increasing the exposure time only below the saturation limit of the detector, thereby preventing saturation and the associated increase in uncorrelated noise (Tornado, 2021). A comparison of the baseline levels before and after oversaturation at the same AMS concentration revealed a baseline shift, suggesting the influence of a secondary factor beyond detector saturation. The authors hypothesize that the baseline shift may be attributable to intrinsic fluorescence or scattering effects caused by the proteins (Gautam et al., 2015), as the VLPs accumulate throughout the process. As expected, spectral preprocessing removed those differences in baseline level; however, the split peak remained present in the spectral data.

### 4.3 Effects of preprocessing operations on Raman spectral data and model transfer

The differences in sensor selectivity and Raman spectral features towards AMS and VLP underscore the importance of individual spectral preprocessing. Attribute-specific preprocessing operations beyond baseline correction and signal smoothing were selected to enhance computational selectivity. All preprocessing operations were specified and applied in a defined sequence to enable the model transfer to the process data.

Prior normalization of the spectra before baseline correction and signal smoothing allows not only for accounting for turbidity effects in the previous precipitation step (Dietrich et al., 2024) but also facilitates model transfer to spectral data obtained at different exposure times, which is the case for the AMS models. A Raman band of the OH-bond of water was used for normalization as neither the analytes nor the background interferes in this spectral region, according to the approach of Sinfield and Monwuba (2014). This strategy was chosen for the AMS models to maintain their applicability across different exposure times and across the earlier process steps of precipitation and wash, where turbidity was observed. To solely account for intensity differences caused by the varying applied exposure times, normalization by exposure time (Rolinger et al., 2023) or standard normal variate (SNV) normalization of already preprocessed spectra (Wei et al., 2022; Vaskó et al., 2024; Wei et al., 2022) have been reported. In this study, however, SNV normalization was omitted since the absolute intensity differences of the major peak—the sulfate band at 980 cm^−1^ (Spinner, 2003; Fontana et al., 2013), which represents a target analyte—would be diminished. For the VLP models, OH-band normalization was not implemented as an additional preprocessing step, as its implementation likely limited model performances, possibly due to introducing a larger error in the relatively smaller intensity ranges of the proteins.

Cropping allows for selecting spectral regions of interest by targeted discarding of the others. A comparison of different, manually selected cropping intervals to systematically improve model performance has been reported by Dietrich et al. (2024), driven by the exclusion of residual baseline variance and impurity- or buffer-related interferences. For AMS monitoring, the predominant sulfate band at 980 cm^−1^ (Spinner, 2003; Fontana et al., 2013) was chosen for the linear regression model, and the edge regions potentially exhibiting unintended variability introduced by prior preprocessing steps were discarded for PLS modeling. Both models reliably predict AMS from off-line and on-line spectral data, but lack prediction accuracy at higher exposure times when oversaturation of the predominant sulfate band is present, attributable to the observed split peak behavior. To refine the PLS model for AMS quantification, VIP was used as a data-driven variable selection technique (Mehmood et al., 2012) for metric-based cropping. Such variable selection techniques aim to minimize the loss of important spectral data while improving model robustness (Andersen and Bro, 2010). In studies dealing with spectral data processing, VIP scores have been used as a spectral region selection criterion (Berry et al., 2015; Santos et al., 2018; Bayer et al., 2020) or simply as feature importance to quantitatively evaluate which spectral regions contribute to PLS models (Kuligowski et al., 2012; Wei et al., 2022; Schiemer et al., 2024; Dietrich et al., 2024). Cropping is typically applied as one of the final preprocessing steps before PLS modeling. For VLP modeling, a pre-cropping approach is presented, describing the manual removal of AMS-associated regions next to the protein-associated region. Pre-cropping was introduced as insufficient baseline correction was observed in the protein-associated region, which could not be removed using alternative baseline correction settings. The authors hypothesize that this effect is again attributable to the much higher sensor sensitivity towards AMS than the proteins. Eventually, preprocessed spectra were manually cropped to only use protein-associated regions for modeling, free from potential interferences from the precipitant. In addition to the already relatively small interval of 147 wavenumbers, a further reduced interval comprising only 19 wavenumbers was also tested. Generally, it is worth noting that variable reduction to such narrow intervals may also remove useful information for prediction.

Both presented PLS-VIP models accommodate precipitant predictions from spectral data exhibiting saturation effects. In ranges without saturation, the continuous predictions of the PLS-VIP models exhibit more noise than those from linear regression or the PLS model, which could be attributed to the smaller spectral range resulting from the spectral cropping, and, consequently, a lower information density. To our knowledge, using spectra with such a saturation-induced split peak behavior for prediction has not been previously reported in the literature. In general, the AMS predictions exhibit much lower fluctuations than the VLP predictions, regardless of which developed PLS model is applied for VLP prediction. In addition to the even lower information density used for model building, this phenomenon can also be attributed to Raman spectroscopy’s inherent sensitivity and selectivity towards proteins.

Another multivariate modeling approach for multi-attribute monitoring from spectral data obtained from a single sensor may be indirect hard modeling regression, which describes the spectrum as a sum of prior parameterized peak functions assigned to individual components. First introduced by Alsmeyer et al. (2004) in combination with Raman spectroscopy, indirect hard modeling has been shown to account for non-linear spectral changes (Kriesten et al., 2008; Meyer-Kirschner et al., 2016). In biopharmaceutical processing, it has been applied for in-line monitoring (Müller et al., 2023) and control (Müller et al., 2024) of fermentation processes; however, its use for multi-attribute monitoring during downstream processing has not yet been reported.

In summary, attribute-specific preprocessing operations were strategically employed beyond baseline correction and signal smoothing to enable model transfer.

## 5 Conclusion and outlook

In conclusion, soft sensors based on Raman spectroscopy and chemometrics were developed and transferred to a filtration-based recovery step of precipitated VLPs for monitoring product accumulation and precipitant depletion. The Raman spectrometer was implemented in an on-line loop in the second membrane stage of the dual-stage CFF setup, and near real-time process data were collected from three CFF experiments with variations in initial product concentration and process parameters.

Through the initial investigation of individual contributions of precipitant and product to the spectral data using stock solutions of the pure components, a substantially higher sensor sensitivity was found for AMS than VLPs. Increasing the exposure time to enhance the sensor’s sensitivity towards VLPs led to the oversaturation of the predominant sulfate band directly related to AMS, which impaired the prediction accuracy for AMS by linear regression. With attribute-specific preprocessing operations next to baseline correction and signal smoothing, namely, normalization and VIP-based cropping, and PLS modeling, we successfully demonstrated model transfer for AMS monitoring despite these detector saturation effects.

For simultaneous VLP monitoring, spectral data were differently preprocessed using a pre-cropping approach before baseline correction and signal smoothing, which effectively improved the spectral appearance, as without, insufficient baseline correction was observed in the protein-associated spectral regions. Even though the larger of the two tested pre-cropping intervals led to more consistent PLS model predictions, the VLP predictions exhibit generally much higher fluctuations than the AMS predictions.

This study highlights that soft sensor selectivity towards target quality attributes is highly dependent on, but also, to some extent, limited by the sensor’s inherent selectivity, although it can be further improved by enhancing the computational selectivity using attribute-specific operations for spectral preprocessing.

## Data Availability

The raw data supporting the conclusions of this article will be made available by the authors, without undue reservation.
