# Supplementary material for: Raman-based PAT for multi-attribute monitoring during VLP recovery by dual-stage CFF: attribute-specific spectral preprocessing for model transfer
Source: Front Bioeng Biotechnol. 2025 Aug 21;13:1631807. doi: 10.3389/fbioe.2025.1631807 (PMC12409638; doi:10.3389/fbioe.2025.1631807)
Supplement: Supplementary file 1 [file DataSheet1.pdf]

Supplementary Material

1 PIPING AND INSTRUMENTATION DIAGRAM OF THE DUAL-STAGE CFF SET-UP

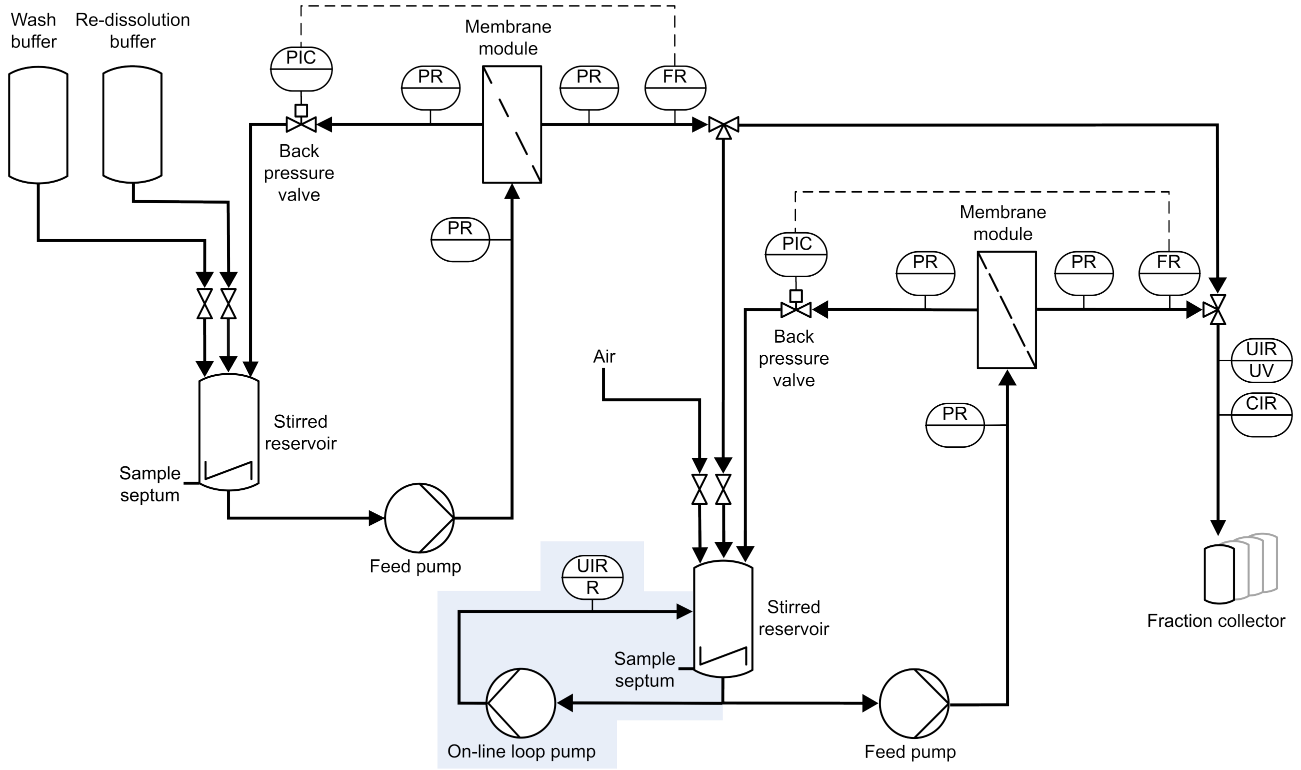

Figure S1. Piping and instrumentation diagram of the dual-stage cross-flow filtration (CFF) set-up. Two serially connected CFF units are equipped with 0.2 µm and 300 kDa MWCO membranes, respectively, and connected to a system for permeate stream fractionation. Starting from the CFF reservoir in the second membrane stage, an on-line monitoring loop was implemented using peek capillaries, a loop pump, and a flow cell for in-line measurements by Raman spectroscopy. The on-line loop is highlighted in blue. C, conductivity or control; F, flow rate; I, indicate; P, pressure; R, record or Raman; U, multivariate; UV, ultraviolet. Adapted from Dietrich et al. (2025).

2 SPECTRAL DATA OF PRECIPITANT-CONTAINING STOCK SOLUTIONS

In general, the spectral appearance of ammonium sulfate (AMS)-containing solutions is due to several precipitant and buffer contributions, as presented in Supplementary Fig. S2. Next to the predominant Raman peak near 980 cm^{-1}, gradually increasing contributions at 450, 618, and 1106 cm^{-1} are also attributable to sulfate ions of AMS, while the bands at 1435 and 1693 cm^{-1} are indicative for ammonium ions (Spinner, 2003; Fontana et al., 2013). On the contrary, bands of buffer components and sapphire remained constant. The bands originating from Tris appear near 1249 and 1470 cm^{-1} (Socrates, 2004). A broad, water-related band is visible near 1650 cm^{-1} (Spinner, 2003), while narrow bands at 379, 418, 430, 450, 577, and 750 cm^{-1} are caused by the sapphire (Watson et al., 1981). Considering spectral interferences, all ammonium bands and the sulfate bands at 450 and 618 cm^{-1} are partially affected

by interference from sapphire or buffer bands, while the sulfate band located at 980 cm^{-1} remained largely unaffected. Further, the contributions of AMS will by far exceed protein-associated contributions in process-derived spectra, mostly located in the wavenumber regions 600–880 cm^{-1}, 1004 cm^{-1}, and 1200–1800 cm^{-1} (Maiti et al., 2004; Rygula et al., 2013).

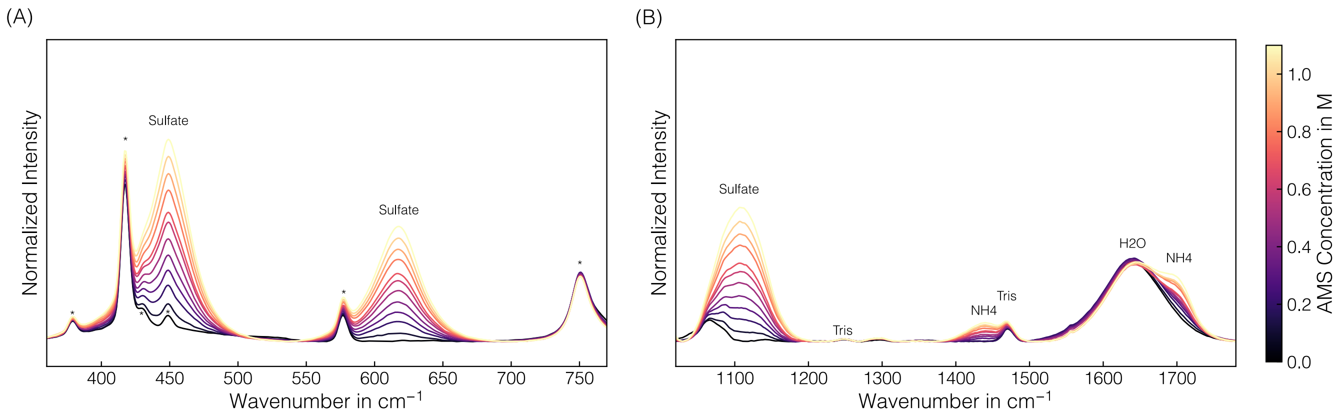

Figure S2. Spectral data comparison. Individual wavenumber intervals of preprocessed Raman spectra derived from a set of stock solutions with varying AMS concentrations are depicted in (A) and (B). Sapphire bands are marked with asterisks, and the other bands were assigned to sulfate ions, ammonium ions, or buffer components. All spectra are shown normalized and colored with brighter colors denoting higher AMS concentrations.

3 DEFECTIVE SPECTRAL DATA

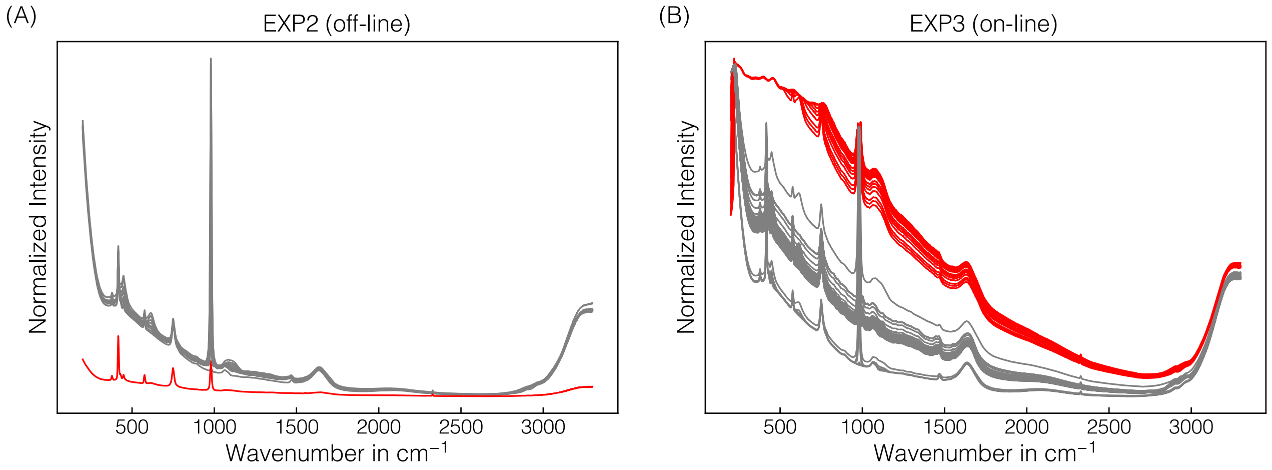

Figure S3. Defective spectra. Raw Raman spectra from EXP2 off-line Raman measurements (A) and EXP3 on-line Raman measurements (B) are shown. Defective spectra are colored in red.

REFERENCES

Dietrich, A., Heim, L., and Hubbuch, J. (2025). Dual-Stage Cross-Flow Filtration: Integrated Capture and Purification of Virus-Like Particles. *Biotechnology and Bioengineering* 122, 884–894. doi:10.1002/bit.28914

Fontana, M. D., Ben Mabrouk, K., and Kauffmann, T. H. (2013). Raman spectroscopic sensors for inorganic salts. In *Spectroscopic Properties of Inorganic and Organometallic Compounds*, eds. J. Yarwood, R. Douthwaite, and S. Duckett (RSC Publishing), vol. 44. 40–67. doi:10.1039/9781849737791-00040

Maiti, N. C., Apetri, M. M., Zagorski, M. G., Carey, P. R., and Anderson, V. E. (2004). Raman Spectroscopic Characterization of Secondary Structure in Natively Unfolded Proteins: α-Synuclein. *Journal of the American Chemical Society* 126, 2399–2408. doi:10.1021/ja0356176

Rygula, A., Majzner, K., Marzec, K. M., Kaczor, A., Pilarczyk, M., and Baranska, M. (2013). Raman spectroscopy of proteins: A review. *Journal of Raman Spectroscopy* 44, 1061–1076. doi:10.1002/jrs.4335

Socrates, G. (2004). *Infrared and Raman Characteristic Group Frequencies: Tables and Charts* (Wiley)

Spinner, E. (2003). Raman-spectral depolarisation ratios of ions in concentrated aqueous solution. The next-to-negligible effect of highly asymmetric ion surroundings on the symmetry properties of polarisability changes during vibrations of symmetric ions. *Spectrochimica Acta Part A: Molecular and Biomolecular Spectroscopy* 59, 1441–1456. doi:10.1016/S1386-1425(02)00293-7

Watson, G. H., Daniels, W. B., and Wang, C. S. (1981). Measurements of Raman intensities and pressure dependence of phonon frequencies in sapphire. *Journal of Applied Physics* 52, 956–958. doi:10.1063/1.328785
